# Evolution of a hotspot genus: geographic variation in speciation and extinction rates in *Banksia* (Proteaceae)

**DOI:** 10.1186/1471-2148-13-155

**Published:** 2013-08-19

**Authors:** Marcel Cardillo, Renae Pratt

**Affiliations:** 1 Macroevolution and Macroecology Group, Research School of Biology, Australian National University, Canberra, ACT 0200, Australia; 2 Current address: Centre for Biodiversity Analysis, Research School of Biology, Australian National University, Canberra, ACT 0200, Australia

**Keywords:** Angiosperm diversity, Diversification, Mediterranean hotspots, Southwest Australia, Species richness

## Abstract

**Background:**

Hotspots of angiosperm species richness and endemism in Mediterranean-climate regions are among the most striking, but least well-understood, geographic patterns of biodiversity. Recent studies have emphasized the importance of rapid diversification within hotspots, compared to non-hotspot regions, as a major contributor to these patterns. We constructed the first near-complete phylogeny of *Banksia* (Proteaceae) to test whether diversification rates have differed between lineages confined to the southwest Australian hotspot and those found throughout southern, eastern and northern Australia. We then tested for variation in diversification rates among the bioclimatic zones within the southwest hotspot itself.

**Results:**

Although *Banksia* species richness in the southwest is ten times that of the rest of the continent, we find little evidence for more rapid diversification in the southwest, although this result is inconclusive. However, we find firmer support for substantial rate variation within the southwest hotspot, with more rapid diversification in the semi-arid heaths and shrublands, compared to the high-rainfall forests. Most of the *Banksia* diversity of the southwest appears to be generated in the heaths and shrublands, with a high migration rate out of this zone boosting diversity of the adjacent forest zone.

**Conclusions:**

The geographic pattern of diversification in *Banksia* appears more complex than can be characterized by a simple hotspot vs. non-hotspot comparison, but in general, these findings contrast with the view that the high diversity of Mediterranean hotspots is underpinned by rapid radiations. Steady accumulation of species at unexceptional rates, but over long periods of time, may also have contributed substantially to the great botanical richness of these regions.

## Background

Mediterranean-climate regions include some of the world’s great repositories of botanical diversity, with levels of angiosperm species richness and endemism that can rival tropical rainforests [1]. However, these environments typically lack features such as high rainfall or productivity that are often associated with high plant diversity on a global scale. Indeed, some of the most species-rich Mediterranean communities are semi-arid heaths and shrublands found on ancient, nutrient-impoverished soils, such as the fynbos of South Africa or the kwongan of southwest Australia [1-3]. Understanding these apparent outliers on global biodiversity gradients may yield insights into the drivers of angiosperm diversification more generally.

A key step in explaining the high botanical diversity of Mediterranean hotspots is demonstrating whether this diversity has arisen as a result of more rapid diversification in these regions, compared to elsewhere, and if so, whether this is the result of a higher rate of speciation or a lower rate of extinction [4]. If diversification rates are not unusually high in Mediterranean hotspots, then alternative explanations for high diversity must be investigated. For example, high diversity may be the result of a steady accumulation of species over a longer period of time, or of higher ecological limits on diversity, in Mediterranean hotspots. However, explicit comparisons of diversification rates between groups within and outside Mediterranean hotspots are rare. Several recent analyses, using very different approaches, support rapid hotspot diversification [5-7]. On the other hand, another study [8] found no evidence for rapid hotspot diversification in an analysis of a large, densely-sampled genus (*Protea*) that spans the boundaries of South Africa’s Cape region.

**Figure 1:**
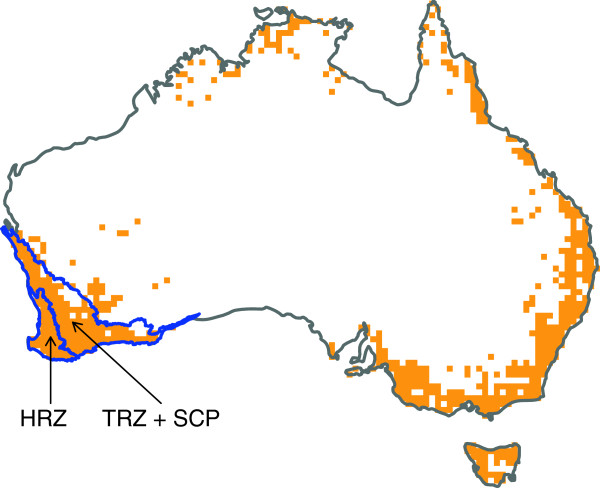
**Map of Australia showing the distribution of *Banksia.*** The overall distribution of the genus is approximated by the locations of origin of herbarium specimens (orange squares). The areas outlined in blue are the transitional rainfall zone and south coast province (TRZ + SCP), and the high rainfall zone (HRZ), which together form the Southwest Botanical Province.

Much of the research effort aimed at understanding Mediterranean plant diversity has focused on the Cape region [3,9]. The angiosperm diversity of Australia’s Southwest Botanical Province (SWBP: Figure 1) is also very impressive (>7300 plant species and 49% endemicity [2]), and is arguably even more intriguing than that of the Cape, because this region has very limited topographic variation. Hopper [2,10] painted a geohistorical picture of the SWBP that explains the region’s high plant diversity in terms of geomorphological and climatic events since the mid-Tertiary. According to this scenario, the widespread formation of lateritic soils during the Oligocene and Miocene, and their subsequent weathering into sandplains, set the scene for a buildup of diversity by providing large areas with a nutrient-poor substrate, for which scleromorphic and xeromorphic taxa were well-adapted. With the onset of increasing aridity during the Tertiary, families such as Myrtaceae and Proteaceae were therefore well-placed for massive diversification [2,10]. In the later Tertiary, uplift of the plateau that covers much of the SWBP led to erosion and dissection of the plateau margins and the creation of a more edaphically-complex landscape. Combined with climate fluctuations during the Quaternary, this may have fragmented species populations, further promoting speciation [2,10].

Hopper [10] acknowledged that geohistorical events alone could not completely explain the high plant diversity of the SWBP. Cowling [11] offered a more deterministic hypothesis based on ecological processes. According to Cowling, frequent fires, a prominent feature of many Mediterranean communities, serve to repeatedly fragment species populations. Combined with poor dispersal capacities and a complex mosaic of soil types, this gives rise to numerous edaphically-specialized populations, and high rates of speciation ensue [11] (see also [12]). This kind of speciation would have become more frequent in the later Tertiary as the climate became drier and the flora became dominated by flammable scleromorphic species, increasing fire frequency [13].

Hopper’s historical scenario and Cowling’s ecological hypothesis both make the simple prediction that diversification rates have been higher in the SWBP compared to less species-rich regions outside the SWBP. A recent broad-scale, genus-level analysis [7] provides some support for this prediction, but it has never been thoroughly tested for any single, well-sampled clade. Furthermore, both hypotheses involve complexities that suggest diversification rates may also vary within the SWBP itself. Within the SWBP, plant diversity tends to be lower in the forested high-rainfall zone (HRZ) of the southwest coastal margin, compared to the heaths and shrublands of the transitional rainfall zone (TRZ) and the southeast coastal province (SCP; [2,10]). Hopper [10] noted that the TRZ and SCP appear to harbour a greater proportion of recently-evolved endemic plant species, suggesting a pattern of more recent, rapid speciation in these regions compared to the HRZ. On the other hand, the proportion of older, “relict” species appears to be higher in the HRZ, suggesting a lower extinction rate in this region. Hopper [2,10] suggested that more complex erosional patterns in the TRZ led to recurrent fragmentation and isolation of populations in the late Tertiary and Quaternary, hence more rapid speciation, while the comparative stability of the lateritic soils in the HRZ favoured larger, more persistent populations, and a lower extinction rate. Under Cowling’s hypothesis, the rate of speciation is linked to the frequency and intensity of fire, both of which vary substantially across different ecosystem types in the SWBP [13]. Hence, this hypothesis also predicts variation in diversification rates within the SWBP.

In this study we use the iconic Australian genus *Banksia* (Proteaceae) as a case study for a comparison of diversification rates (1) between the SWBP and elsewhere in Australia, and (2) between the bioclimatic zones within the SWBP. Based on molecular evidence [14], a recent taxonomic revision [15] sunk the former genus *Dryandra* into *Banksia*, forming a large new genus of 170 species. The majority of these are endemic to the SWBP, but *Banksia* have dispersed throughout the southern, eastern and northern parts of Australia (Figure 1). We present the first near-complete species-level molecular phylogeny of *Banksia sensu lato*. We then use this phylogeny to reconstruct the ancestral range of *Banksia,* and to compare rates of diversification between regions, using recently-developed methods that allow for reciprocal effects of diversification and shifts in geographic distribution [16].

## Methods

### Sampling, DNA extraction and sequencing

We used a combination of previously-published sequences for 88 *Banksia* taxa (including multiple subspecies within some species)*,* and newly-generated sequence data for 110 taxa. Tissue samples were collected from dried specimens held at the Australian National Herbarium, Canberra, and from living plants at the Banksia Farm arboretum, Mt. Barker, Western Australia. We extracted total genomic DNA using a Qiagen DNEasy Plant Mini Kit according to the manufacturer’s protocol (Qiagen Inc., California, USA). We amplified DNA from four noncoding regions of the chloroplast genome; 994bp of the *trnL-trnF* intergenic spacer, 1113bp of the *rpl16* intron, 578bp of the *psbA/trnH* spacer and 873bp of the *trnT-trnL* spacer. Sequencing was carried out at Macrogen on a 3730XL DNA sequencer (Macrogen Inc., Seoul, Korea) using the same primers used to amplify the product. Details of primer sequences and GenBank accessions are provided in Additional files 1 and 2.

### Phylogeny reconstruction and dating

We concatenated published and newly-generated sequences from the four cpDNA markers for 198 *Banksia* taxa, together with sequences from GenBank for six outgroup species (*Panopsis costaricensis*, *Musgravea heterophylla*, *Grevillea renwickiana*, *Grevillea scortechinii*, *Macadamia integrifolia* and *Hakea victoria*). Sequences were aligned using automated alignment followed by manual editing in Geneious [17]. The phylogeny was estimated together with divergence times using a Bayesian analysis in BEAST 1.6.2 [18]. Calibrations were based on ages estimated from fossil data [19]; this reference gives details of the fossils used and the method by which stratigraphic ages were converted to absolute time estimates. We placed lognormal priors on the ages (in millions of years) of the *Banksia* crown node (mean = 42, sd = 0.4) and the *Banksia* stem node (mean = 62, sd = 0.4). Because the formerly separate genus *Dryandra* is now believed to be nested within *Banksia*, our *Banksia* stem node corresponds to the node considered to be the stem of the tribe Banksieae by Crisp & Cook [19], for which they assumed a minimum age of 62Mya. We used lognormal priors for these calibrations because this allows for uncertainty around the age estimates, while still reflecting the belief that these ages represent minimum bounds on the estimated divergence times [20]. For the root node we used a normal prior (mean = 77, sd = 1), based on the estimated divergence time between the lineages leading to *Banksia* and *Hakea / Grevillea* in a comprehensive genus-level phylogeny of the Proteaceae [7]. Because this was a secondary calibration that does not represent a minimum bound, we felt that a normally-distributed prior was most appropriate. We used a Yule prior for the speciation process, with an uncorrelated lognormal model of variation in rate of molecular evolution. The function modelTest in the R library phangorn v.1.6.0 [21] was used to select an HKY + gamma substitution model. We ran two separate MCMC runs for >40,000,000 generations, sampling trees every 10,000 generations. We diagnosed sampling adequacy by examining the effective sample sizes (ESS) using Tracer 1.5 [22], and stopped the runs when the parameter values of the combined runs all had ESS > 300.

Our phylogenies included multiple subspecies or varieties for some species. To avoid biasing our analyses by the variable representation of intraspecific taxa, we pruned the trees to the species level before all analyses. Each time a new tree was called from the posterior distribution, we randomly sampled one tip from each species represented by multiple tips.

### Assigning species to geographic regions

Geographic range maps were constructed for each species by obtaining all *Banksia* and *Dryandra* collection records from the Australian Virtual Herbarium (http://chah.gov.au/avh/), then removing records that were clearly outside the natural distribution limits of each species (e.g. those from botanic gardens in capital cities) by checking each map against published maps in atlases of *Banksia*[23] and *Dryandra*[24] species. We first classified each species as belonging to the SWBP or not. This was relatively straightforward as distributions of southwestern Australian *Banksia* are clearly separated from the rest of Australia by arid barriers to the north and east, and no species has a distribution than spans these barriers. The distributions of a few species are slightly outside the formal boundary of the SWBP, but still clearly separated from the eastern and northern Australian species – these species were assigned to the SWBP. We classified each SWBP species as belonging to the HRZ, the TRZ/SCP, or both. This was done by overlaying each species distribution onto a map of the rainfall zone boundaries and counting the number of records within each zone. If >90% of a species’ records fell in one zone, the species was assigned to this zone; otherwise the zone was recorded as “both”. All mapping was done using the maptools, rgdal, and raster libraries in R. The numbers of species of each geographic state that were present / not present in the phylogeny are as follows. non-SWBP: 15/1; SWBP: 143/12; HRZ: 11/0; TRZ+SCP: 78/9; both HRZ and TRZ+SCP: 54/3.

We then reconstructed the ancestral geographic range of all nodes in the *Banksia* tree as a binary variable (SWBP/non-SWBP) by maximum likelihood, using the Mk2 model in the R library diversitree.

### Geographic variation in diversification rates

To compare rates of diversification among regions, and rates of migration across regions, we used two related methods. First, we tested whether diversification varies between SWBP and non-SWBP species using BiSSE [25], implemented in diversitree. The BiSSE method implements maximum likelihood estimates of six parameters under a constant-rates birth-death process: speciation and extinction rates under each of the two states of a binary character (λ_0_, λ_1_, μ_0_, μ_1_), rates of transition from state zero to state one (m_01_), and rates of transition from state one to state zero (m_10_). In our analyses, we treated SWBP and non-SWBP species as displaying state zero and state one, respectively. Second, we tested whether diversification rates vary among the rainfall zones within the SWBP, using GeoSSE [16]. GeoSSE differs from BiSSE by allowing for reciprocal effects of geographic distribution and diversification rates; for example, a species distributed across two geographic regions may speciate to produce two daughter species each with a distribution restricted to a single region. In addition to the six BiSSE parameters, therefore, GeoSSE estimates a seventh parameter for between-region speciation. For this test we compared species belonging to the HRZ with those belonging to the TRZ/SCP. Because the GeoSSE model requires that all three geographic states (region A, region B, and both) be occupied by at least some species, we could not apply GeoSSE to the SWBP/non-SWBP comparison, since no species are distributed across the SWBP boundary. For this comparison we used BiSSE, which does not have this restriction. For both BiSSE and GeoSSE, we specified the proportion of species in each geographic zone that are not present in the phylogeny.

To test for variation in diversification and migration rates between SWBP and non-SWBP species, we began by fitting a BiSSE model in which all six parameters were allowed to vary (full model). We then fitted three constrained models in which (1) speciation and extinction rates were held equal across regions, and migration from and to the SWBP were held equal (equal-rates model); (2) speciation and migration were held equal but extinction allowed to vary (equal-speciation model); and (3) extinction and migration were held equal but speciation allowed to vary (equal-extinction model). We did not fit a model that explicitly tests the influence of varying migration rates while holding speciation and extinction constant, because migration rates into and out of the SWBP are likely to have been too low to have any important influence on differences in species richness. We compared the fit of the four models to the data using AIC. All tests were repeated for 250 phylogenies sampled evenly from the Bayesian posterior distribution; we felt that this number was sufficient to adequately represent the variation among trees within the posterior set.

To test for variation in diversification and migration rates among rainfall zones within the SWBP, we used GeoSSE rather than BiSSE. In this set of tests we were explicitly interested in the role of migration rates between rainfall zones, so we tested the following five models: (1) all six parameters allowed to vary (full model); (2) all parameters held equal (equal-rates model); (3) speciation held equal but extinction and migration allowed to vary (equal-speciation model); (4) extinction held equal but speciation and migration allowed to vary (equal-extinction model); (5) migration held equal but speciation and extinction allowed to vary (equal-migration model). As above, we compared models using AIC and repeated tests on the same sample of 250 phylogenies as the BiSSE analysis.

## Results

### Banksia phylogeny and ancestral region

**Figure 2:**
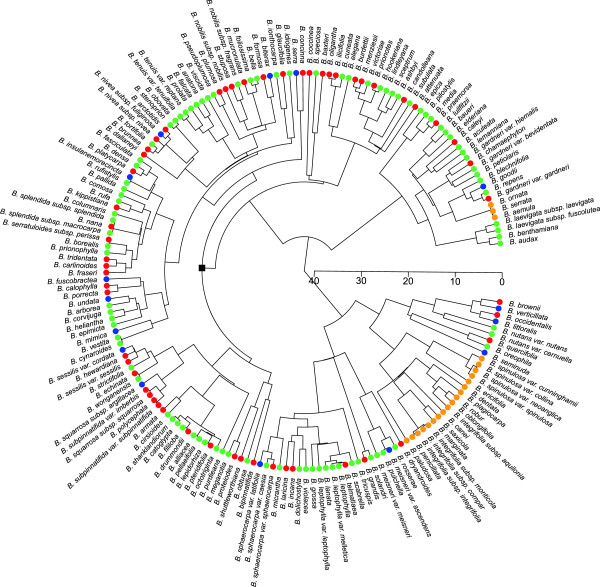
**Maximum clade credibility tree of *Banksia *from a BEAST analysis.** Coloured symbols at the tips indicate geographic distributions: orange = non-SWBP; red = TRZ + SCP; blue = HRZ; green = both TRZ + SCP and HRZ. The black square indicates the crown node of the Dryandra clade. Timescale is in millions of years bp.

The Bayesian phylogenies recovered from chloroplast data show that *Banksia* and *Dryandra* are not sister taxa, but that the Dryandra clade is a large and relatively recent radiation within *Banksia* (Figure 2), supporting the transfer of *Dryandra* to *Banksia*[15]*.* The phylogenies also support the division of *Banksia* into two major clades, *Banksia* subgenus *Banksia* and *Banksia* subgenus *Spathulatae*[15]. Most nodes are well-supported by the Bayesian analysis, but there is poor resolution within the Dryandra clade. All non-SWBP species are grouped into two distantly-related clades, suggesting two independent migration events out of the SWBP. This is supported by the reconstruction of the ancestral region of *Banksia*: there is a median probability of 0.993 (across the 250 sampled trees) that the geographic state at the crown node of *Banksia* was the SWBP. A nexus file of the maximum clade credibility tree, showing median node heights, likelihoods and posterior support values, is provided in Additional file 3. The set of 250 sampled phylogenies used in the analyses is provided in Additional file 4.

### Temporal patterns of diversification

**Figure 3:**
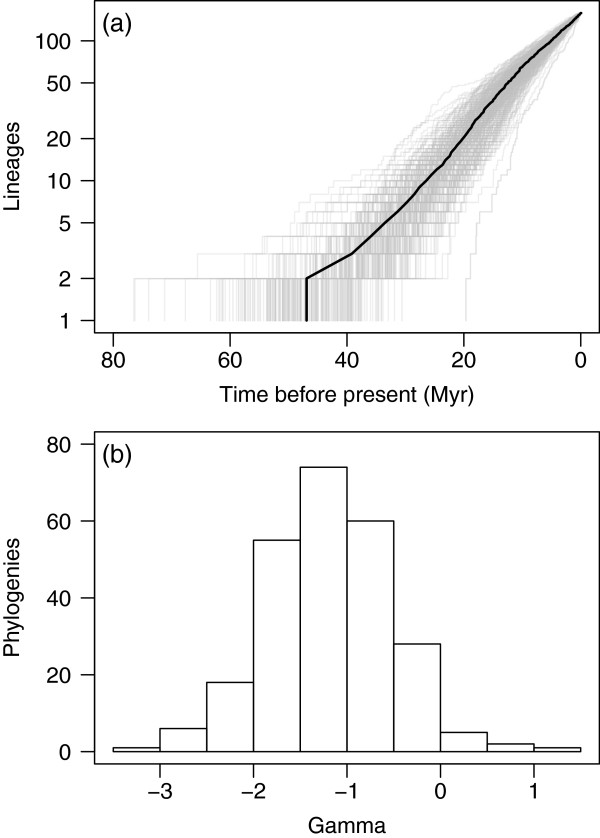
**Temporal patterns of *Banksia *diversification. (a)** Accumulation of lineages through time for a sample of 250 *Banksia* phylogenies from the Bayesian posterior distribution. The solid line connects the median divergence times for each number of lineages. **(b)** Distribution of values of the gamma statistic for the 250 phylogenies.

**Figure 4:**
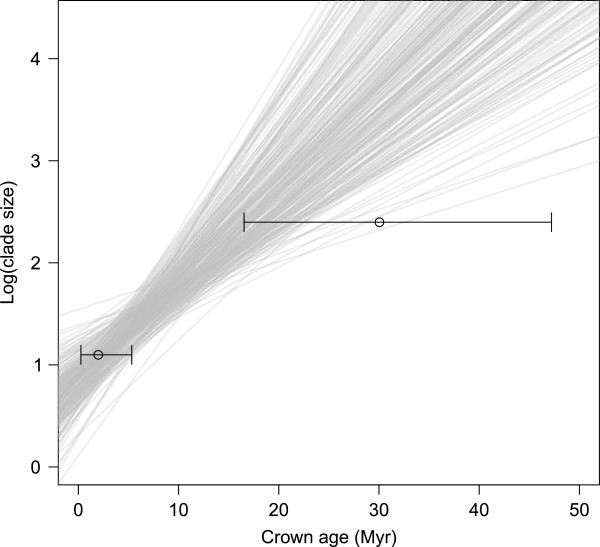
**Regressions of crown age against clade size.** For each of 250 *Banksia* phylogenies sampled from the Bayesian posterior distribution, a set of independent clades was identified by counting six nodes down from the root. Regressions were fitted across these clades, giving 250 slope estimates. The open circles and bars represent the median and HPD of the crown age estimates for the two non-SWBP *Banksia* clades.

Overall, *Banksia* appears to have diversified by a steady accumulation of species rather than a rapid radiation. The semilogarithmic plot of lineages through time (Figure 3a) approximates a straight line for most trees, indicating a constant rate of diversification, although there is evidence for a slight slowdown in diversification rates after 20 Myr ago. This slight slowdown produces negative values of gamma [26] in the majority of the 250 sampled phylogenies (Figure 3b), although only 30 (12%) deviate significantly from the expectation under a constant-rates model. There is a strong positive association between the crown ages of clades and their species richness in all of the sampled trees (Figure 4), as expected under a constant-rates model of diversification. The median of the estimated crown ages of the smaller of the two non-SWBP clades (the lower circle in Figure 4) is well within that expected for its species richness. For the larger of the non-SWBP clades, the median crown age estimate lies in the upper tail of the distribution of expected values (the upper circle in Figure 4). While this might indicate unusually slow diversification in this clade compared to other *Banksia* clades of similar age, the 95% highest posterior distribution (HPD) of the crown age estimates largely overlaps the distribution of expected values, so this cannot be considered a significant departure from the expected distribution.

### Geographic patterns of diversification

**Figure 5:**
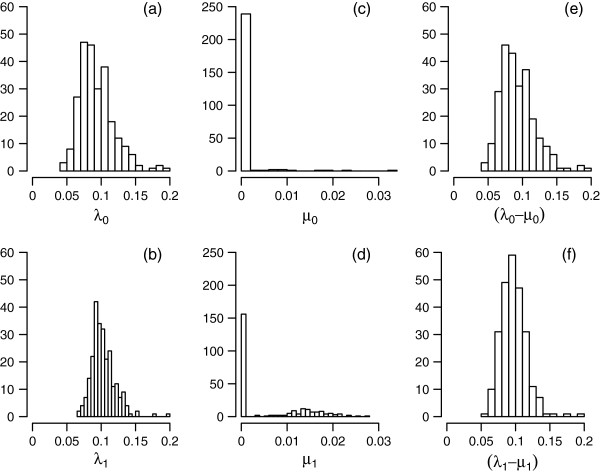
**Distributions of BiSSE parameter estimates.** The panels show distributions of estimates of **(a,b)** speciation rates, **(c,d)** extinction rates, and **(e,f)** diversification rates, for 250 phylogenies from the Bayesian posterior distribution, from a BiSSE analysis in which all parameters were free to vary (the full model). Upper and lower panels are estimates for non-SWBP and SWBP clades, respectively.

**Figure 6:**
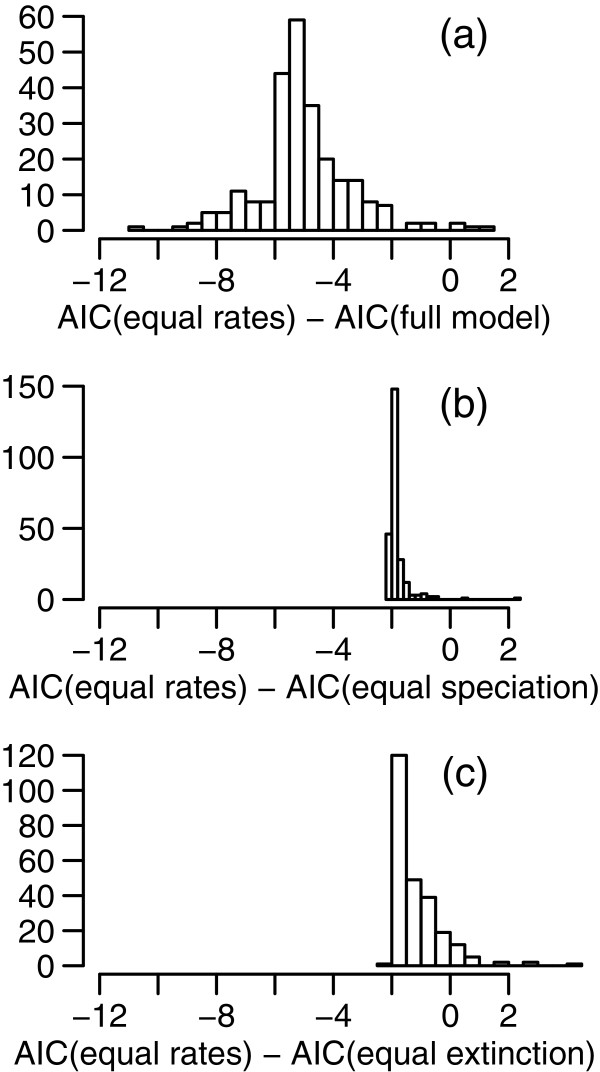
**BiSSE model comparisons.** The panels show ∆AIC values for 250 phylogenies from the Bayesian posterior distribution. **(a)** Equal-rates vs full model; **(b)** Equal-rates vs equal-speciation model; **(c)** equal-rates vs equal-extinction model.

The BiSSE analysis offers no evidence that speciation or extinction rates differ between SWBP and non-SWBP *Banksia* clades. Figure 5 shows the distributions of estimates of speciation rates, extinction rates, and net diversification rates, from the full model (in which all parameters are permitted to vary). Distributions of speciation rates are very similar for SWBP and non-SWBP clades, with median values of 0.1 and 0.09, respectively, across the 250 sampled trees. Extinction rates are estimated at zero in the majority of trees, with positive extinction estimates in a small number of trees. Comparisons of models using AIC (Figure 6) indicate strongest support for the equal-rates model. The equal-rates model provides substantially better fit to the data than the full model, with differences in AIC (∆AIC) greater than 2 for the majority of trees (Figure 6a). The equal-rates model is also a slightly better fit than the equal-speciation and equal-extinction models in 97.2% phylogenies, although ΔAIC values are nearly all less than 2 (Figure 6b, 6c).

**Figure 7:**
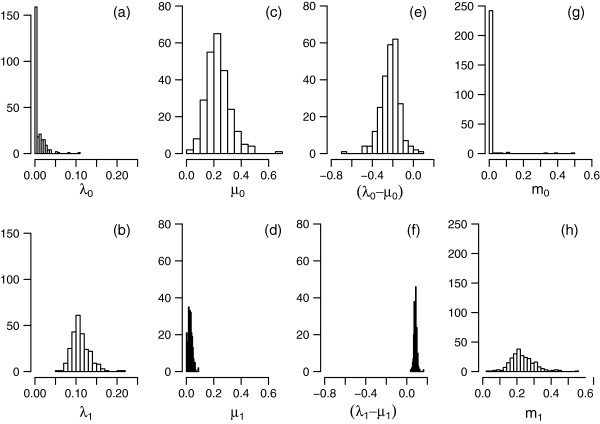
**Distributions of GeoSSE parameter estimates.** The panels show distributions of estimates of **(a,b)** speciation rates, **(c,d)** extinction rates, **(e,f)** diversification rates, and **(g,h)** migration rates, for 250 phylogenies from the Bayesian posterior distribution, from a GeoSSE analysis in which all parameters were free to vary (the full model). Upper and lower panels are estimates for HRZ and TRZ + SCP species, respectively.

**Figure 8:**
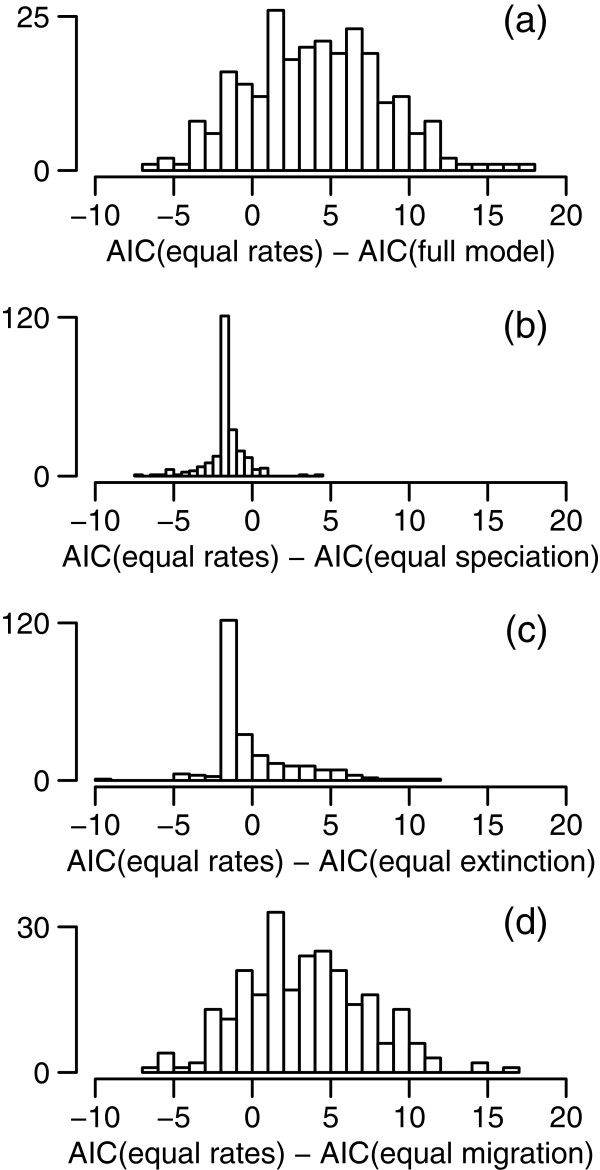
**GeoSSE model comparisons.** The panels show ∆AIC values for 250 phylogenies from the Bayesian posterior distribution. **(a)** Equal-rates vs full model; **(b)** Equal-rates vs equal-speciation model; **(c)** equal-rates vs equal-extinction model; **(d)** equal-rates vs equal-dispersal model.

On the other hand, the GeoSSE analysis reveals differences in speciation, extinction, and migration rates among the different rainfall zones within the SWBP. Figure 7 shows the distributions of estimates of speciation rates, extinction rates, net diversification rates, and migration rates, from the full model. Median speciation rate estimates across the 250 trees are 0.11 in TRZ+SCP and 0 in HRZ clades (Figure 7a, 7b). Median extinction rate estimates are 0.03 and 0.22 in TRZ+SCP and HRZ clades, respectively (Figure 7c, 7d). For many of the HRZ phylogenies, extinction rates that are higher than speciation rates give rise to negative net diversification rates (Figure 7e). This seems implausible for phylogenies reconstructed from extant species, and may be the result of imprecision in the extinction rate estimates [27]. Median migration rate estimates are 0.22 for migration out of the TRZ+SCP, and 0 for migration into the TRZ+SCP (Figure 7e, 7f). Model comparisons (Figure 8) indicate strongest support for the full model, in which all parameters are allowed to vary. The full model is more strongly supported than the equal-rates model, with ΔAIC > 2 in 66% of trees (Figure 8a), but the majority of phylogenies show little difference between equal-rates and equal-speciation models (Figure 8b), or between equal-rates and equal-extinction models (Figure 8c). The equal-rates model is also a better fit than the equal-migration model in a slight majority (59.2%) of phylogenies (Figure 8d).

## Discussion

Although the nearest extant relatives of *Banksia* (*Austromuellera* and *Musgravea*) are confined to the rainforests of northeastern Queensland, at the opposite end of the Australian continent, our reconstruction of ancestral regions places the crown node of *Banksia* in the SWBP with a high probability. It seems likely, therefore, that *Banksia* began diversifying within the SWBP, and that the extant non-SWBP species of *Banksia* are derived from two independent dispersal events out of the southwest and throughout northern, southern and eastern Australia. One important issue relevant to understanding the high floristic diversity and endemism of the SWBP hotspot is the role played by the Nullarbor Plain in driving divergences between eastern and western species. The Nullarbor Plain is an arid limestone plateau that developed around 13–14 Myr ago, potentially acting as both an aridity barrier and an edaphic barrier to the east–west dispersal of plant species [19]. Crisp & Cook [19] found that divergences between eastern and western Australian clades within a number of plant genera seemed to cluster around 13–14 Myr ago, leading them to conclude that the Nullarbor Plain played a significant role in the isolation of the SWBP flora. Their reconstructed divergence time for the smaller of the two eastern *Banksia* clades was 16.3 Myr (CI = 4.3 – 31.3Myr), based on the split between *B. benthamiana* and *B. serrata*. However, our phylogeny reveals several SWBP species more closely-related to *B. serrata* than *B. benthamiana*, giving a median estimate for the divergence of this clade from its SWBP sister clade of only 8.1 Myr (95% HPD = 3.2 – 13.3 Myr), post-dating the formation of the Nullarbor Plain. This suggests that while the aridity and edaphic barrier formed by the Nullarbor Plain may have restricted the frequency of east–west dispersal of *Banksia*, it is unlikely to have been a hard barrier to dispersal.

Our BiSSE analysis found no evidence that the two dispersal events out of the southwest corresponded either with a reduced speciation rate or with an increased extinction rate. One possible explanation for this is a simple lack of statistical power. A recent simulation study [27] showed that the BiSSE method has relatively low power to detect rate differences among states of a binary trait in phylogenies with fewer than 300 tips and a high degree of asymmetry in tip states. Our *Banksia* phylogenies have 158 tips of which 15 (9.5%) have state zero (i.e. non-SWBP). The results of our BiSSE analysis must therefore be regarded as inconclusive, and treated cautiously. Indeed, the position of the larger non-SWBP clade on the plot of clade age x log(species richness) (Figure 4) hints at the possibility that migration out of the SWBP was accompanied by reduced diversification rates, although again, this result is inconclusive.

The expansion of *Banksia* out of the southwest opened up vast new areas of the Australian continent in which the genus could diversify. Regardless of whether diversification rates remained steady or were reduced following this expansion, it appears that the expansion out of the southwest did not provide significant new ecological opportunities for diversification. The difference in mean geographic range size between SWBP and non-SWBP *Banksia* species supports this view. In the SWBP, geographically fine-scale speciation has produced numerous narrowly-distributed species, whereas the non-SWBP clades are characterized by fewer, more widely-distributed species. Hence, although non-SWBP clades occupy a collective area many times larger than the SWBP, species density is far lower. In this respect, the situation for *Banksia* is intriguingly similar to that of another hotspot genus, the African *Protea. Protea* is a large genus of Proteaceae with the great majority of its species endemic to the Cape biodiversity hotspot, but with a small number species, derived from only a single migration event out of the Cape, distributed across a large area of Africa [8]. Like *Banksia*, the non-hotspot species of *Protea* tend to occupy far larger geographic ranges than the hotspot species, and Valente *et al.*[8] found no evidence for a difference in diversification rates between Cape and non-Cape clades (although the above caveat about the statistical power of BiSSE also applies to their analysis). The similarity of patterns between *Banksia* and *Protea* suggests a possible common mechanism driving diversification in the two genera. One possibility is that strong habitat affinities (niche conservatism) and poor dispersal capabilities limited migration out of the hotspot regions. Successful migration may have been achieved only rarely, perhaps by chance, or perhaps by unusually good dispersers which then gave rise to clades of descendants that inherited the traits associated with high dispersal ability. Unfortunately, this hypothesis will be difficult to test in *Banksia* until good data on dispersal ability (e.g. [28,29] become available for many species. Even then, with only two comparisons it would be difficult to support this hypothesis to the exclusion of possible confounding factors.

While we found little firm evidence that diversification rates differ between SWBP and non-SWBP clades of *Banksia*, the GeoSSE analysis revealed differences in rates among the rainfall zones within the SWBP itself. In the transitional rainfall zone (TRZ) and south coast province (SCP), speciation rates are higher, and extinction rates lower, than in the high rainfall zone (HRZ). In addition, rates of migration from the TRZ/SCP to the HRZ were higher than in the opposite direction. These results point to a scenario where the sandplain heaths and shrublands of the TRZ/SCP act as a “biodiversity pump”, responsible for generating much of the *Banksia* diversity of the SWBP, with diversity of the adjacent coastal forests of the HRZ elevated by migration out of the TRZ/SCP. This scenario is at least partly consistent with Hopper’s geohistorical hypothesis [2,10]. Under Hopper’s hypothesis, high speciation rates in the TRZ/SCP resulted from increasing landscape complexity in the later part of the Tertiary Period, following a long period of relative stability. The resulting fragmentation of populations may have led to widespread allopatric speciation and the formation of numerous narrowly-distributed endemic species. However, this scenario does not account for the reduced extinction rates in the TRZ/SCP, compared to the HRZ, that the GeoSSE analysis indicates. Indeed, Hopper suggested that the relative stability of HRZ landscapes should have led to lower extinction rates there, and the accumulation of “relict” species. One possibility is that regional-scale variation in fire frequency and intensity have played an important role in regulating extinction rates. If fires were more frequent and intense in the densely-vegetated sclerophyll forests, compared to the more sparsely-vegetated heaths and shrublands, extinction resulting from population fragmentation may have been more frequent in the HRZ. There is some evidence that the forests have been more fire-prone than the heaths and shrublands over the comparatively recent period of human occupation of southwestern Australia [30], but there are little data with which to infer geographic variation in fire patterns across the region over the deeper timescales relevant to macroevolution [13].

## Conclusions

Mediterranean-climate hotspots of botanical diversity are among the most striking, but least well-understood, of the world’s large-scale biodiversity patterns. We have found little evidence that the diversity of *Banksia*, one of the largest genera in the southwest Australian hotspot, is the result of anything other than a steady accumulation of species over a long period of time. This contrasts markedly with the view that the great diversity of Mediterranean hotspots is underpinned by rapid radiations of plant genera [5,7]. There are, of course, other genera (such as *Hakea*) that appear to have radiated much more rapidly than *Banksia*[7], and also have their greatest diversity in the SWBP. However, there can be no basis for assuming that diversification in *Hakea* and other genera has been more rapid in the hotspot region, until comparisons of diversification rates across the hotspot boundaries, similar to those we have presented here, are made. In order to understand the relative contribution of rapid radiations versus steady diversification to the botanical diversity of Mediterranean hotspots, therefore, it will be necessary to produce and analyse densely-sampled phylogenies of multiple hotspot genera.

### Availability of supporting data

The data sets supporting this article are included within the article and its additional files.

DNA sequence data have been deposited with GenBank (http://www.ncbi.nlm.nih.gov/genbank/). Accession numbers are provided in Additional file 1.

The full posterior set of Banksia phylogenies from the BEAST analysis will be deposited in the Data Dryad repository (http://datadryad.org/) upon acceptance of the manuscript for publication.

## Competing interests

The authors declare that they have no competing interests.

## Authors’ contributions

RP performed the DNA extractions and other laboratory work, did the initial sequence alignments and preliminary phylogenetic analyses, and edited the manuscript. MC designed the study, carried out the fieldwork, performed the analyses and wrote the manuscript. Both authors read and approved the final manuscript.

## Supplementary Material

Additional file 1 **GenBank accession numbers for *Banksia *taxa included in the study.** Click here for file

Additional file 2 Chloroplast regions amplified and primer sequences. Click here for file

Additional file 3 Nexus file of the maximum clade credibility tree from the BEAST analysis. Click here for file

Additional file 4 Phylip file of the 250 phylogenies sampled from the BEAST posterior distribution. Click here for file
