# Supplementary material for: Evolution of a hotspot genus: geographic variation in speciation and extinction rates in Banksia (Proteaceae)
Source: BMC Evol Biol. 2013 Aug 19;13:155. doi: 10.1186/1471-2148-13-155 (PMC3751403; doi:10.1186/1471-2148-13-155)
Supplement: Additional file 1 — GenBank accession numbers for Banksia taxa included in the study. [file 1471-2148-13-155-S1.docx]

**Additional File 1. GenBank accession numbers for *Banksia* taxa included in the study**

Cardillo, M. & Pratt, R. Evolution of a hotspot genus: geographic variation in speciation and extinction rates in *Banksia* (Proteaceae)

| Taxon | | Chloroplast DNA region | | | | |
| --- | --- | --- | --- | --- | --- | --- |
| Previous name | Current name | *trnL* intron | *trnL-trnF* intergenic spacer | *rpl16* intron | *psbA-trnH* spacer | *trnT-trnL* spacer |
| *Banksia aculeata* | *Banksia aculeata* |  |  | AF482202 | AF482022 | AF482303 |
| *Banksia aemula* | *Banksia aemula* | AF482110 | AF482155 | AF482206 | AF482026 | AF482307 |
| *Banksia ashbyi* | *Banksia ashbyi* | AF482119 | AF482164 | AF482219 | AF482039 | AF482320 |
| *Banksia attenuata* | *Banksia attenuata* | AF482118 | AF482163 | AF482218 | AF482038 | AF482319 |
| *Banksia audax* | *Banksia audax* |  |  | AF482233 | AF482053 | AF482334 |
| *Banksia baueri* | *Banksia baueri* | AF482116 | AF482161 | AF482216 | AF482036 | AF482317 |
| *Banksia baxteri* | *Banksia baxteri* | AF482113 | AF482158 | AF482209 | AF482029 | AF482310 |
| *Banksia benthamia* | *Banksia benthamiana* | AF482123 | AF482168 | AF482232 | AF482052 | AF482333 |
| *Banksia blechnifolia* | *Banksia blechnifolia* |  |  | AF482224 | AF482044 | AF482325 |
| *Banksia brownii* | *Banksia brownii* | AF482127 | AF482172 | AF482255 | AF482075 |  |
| *Banksia burdettii* | *Banksia burdettii* |  |  | AF482212 |  | AF482313 |
| *Banksia caleyi* | *Banksia caleyi* |  |  | AF482201 | AF482021 | AF482302 |
| *Banksia candollea* | *Banksia candollea* | AF482111 | AF482156 | AF482207 | AF482027 | AF482308 |
| *Banksia canei* | *Banksia canei* |  |  | AF482243 | AF482063 |  |
| *Banksia chamaephyton* | *Banksia chamaephyton* |  |  | AF482223 | AF482043 | AF482324 |
| *Banksia coccinea* | *Banksia coccinea* | AF482120 | AF482165 | AF482220 | AF482040 | AF482321 |
| *Banksia dentata* | *Banksia dentata* |  |  | AF482240 | AF482060 |  |
| *Banksia dolichostyla* | *Banksia sphaerocarpa var. dolichostyla* |  |  | AF482265 | AF482085 |  |
| *Banksia dryandroides* | *Banksia dryandroides* | AF482129 | AF482174 | AF482261 | AF482081 |  |
| *Banksia elderiana* | *Banksia elderiana* | AF482108 | AF482153 | AF482199 | AF482019 | AF482300 |
| *Banksia elegans* | *Banksia elegans* | AF482107 | AF482152 | AF482198 | AF482018 | AF482299 |
| *Banksia epica* | *Banksia epica* |  |  | AF482230 | AF482050 | AF482331 |
| *Banksia ericifolia* | *Banksia ericifolia* | AF482126 | AF482171 | AF482253 | AF482073 |  |
| *Banksia gardneri var. bevidentata* | *Banksia gardneri var. brevidentata* |  | JQ765201 | JQ765031 | JQ765284, AF482046 | AF482327 |
| *Banksia gardneri var. gardneri* | *Banksia gardneri var. gardneri* |  |  | JQ765032 | JQ765374 | JQ765128 |
| *Banksia gardneri var. hiemalis* | *Banksia gardneri var. hiemalis* |  | JQ765202 | JQ765033 | JQ765313 | JQ765130, AF482326 |
| *Banksia goodii* | *Banksia goodii* |  |  | AF482227 | AF482047 | AF482328 |
| *Banksia grandis* | *Banksia grandis* | AF482124 | AF482169 | AF482235 | AF482055 |  |
| *Banksia grossa* | *Banksia grossa* |  |  | AF482264 | AF482084 |  |
| *Banksia hiemalis* | *Banksia gardneri var. hiemalis* |  |  |  | JQ765312 |  |
| *Banksia hookeria* | *Banksia hookeriana* |  |  | AF482214 | AF482034 | AF482315 |
| *Banksia ilicifolia* | *Banksia ilicifolia* | AF482105 | AF482150 | AF482195 | AF482015 | AF482296 |
| *Banksia incana* | *Banksia incana* |  |  | AF482276 | AF482096 |  |
| *Banksia integrifolia subsp. aquilonia* | *Banksia aquilonia* |  |  | AF482248 | AF482068 |  |
| *Banksia integrifolia subsp. compar* | *Banksia integrifolia subsp. compar* |  |  | AF482246 | AF482066 |  |
| *Banksia integrifolia subsp. integrifolia* | *Banksia integrifolia subsp. integrifolia* |  |  | AF482245 | AF482065 |  |
| *Banksia integrifolia subsp. monticola* | *Banksia integrifolia subsp. monticola* |  |  | AF482247 | AF482067 |  |
| *Banksia laevigata subsp. fuscolutea* | *Banksia laevigata subsp. fuscolutea* |  | JQ765208 | JQ765040 | JQ765320 | JQ765134 |
| *Banksia laevigata subsp. laevigata* | *Banksia laevigata subsp. laevigata* |  |  | AF482234 | AF482054 | AF482335 |
| *Banksia lanata* | *Banksia lanata* |  |  | AF482273 | AF482093 |  |
| *Banksia laricina* | *Banksia laricina* |  |  | AF482275 | AF482095 |  |
| *Banksia lemanniana* | *Banksia lemanniana* |  |  | AF482200 | AF482020 | AF482301 |
| *Banksia leptophylla var. leptophylla* | *Banksia leptophylla var. leptophylla* |  |  | AF482272 | AF482092 |  |
| *Banksia leptophylla var. melletica* | *Banksia leptophylla var. melletica* |  |  | AF482271 | AF482091 |  |
| *Banksia lindleyana* | *Banksia lindleyana* | AF482109 | AF482154 | AF482203 | AF482023 | AF482304 |
| *Banksia littoralis* | *Banksia littoralis* |  |  | AF482258 | AF482078 |  |
| *Banksia lullfitzii* | *Banksia lullfitzii* | AF482117 | AF482162 | AF482217 | AF482037 | AF482318 |
| *Banksia marginata* | *Banksia marginata* |  |  | AF482241 | AF482061 |  |
| *Banksia media* | *Banksia media* | AF482122 | AF482167 | AF482229 | AF482049 | AF482330 |
| *Banksia meisneri subsp. meisneri* | *Banksia meisneri var. meisneri* |  | JQ765260 |  |  |  |
| *Banksia meisneri var. ascendens* | *Banksia meisneri subsp. ascendens* |  |  | AF482279 | AF482099 |  |
| *Banksia menziesii* | *Banksia menziesii* | AF482115 | AF482160 | AF482211 | AF482031 | AF482312 |
| *Banksia micrantha* | *Banksia micrantha* |  |  | AF482266 | AF482086 |  |
| *Banksia nutans var. cernuella* | *Banksia nutans var. cernuella* | AF482130 | AF482175 | AF482263 | AF482083 |  |
| *Banksia nutans var. nutans* | *Banksia nutans var. nutans* |  |  | AF482262 | AF482082 |  |
| *Banksia oblongifolia* | *Banksia oblongifolia* | AF482125 | AF482170 | AF482237 | AF482057 |  |
| *Banksia occidentalis* | *Banksia occidentalis* |  |  | AF482254 | AF482074 |  |
| *Banksia oligantha* | *Banksia oligantha* |  |  | AF482196 | AF482016 | AF482297 |
| *Banksia oreophila* | *Banksia oreophila* |  |  | AF482260 | AF482080 |  |
| *Banksia ornata* | *Banksia ornata* |  |  | AF482204 | AF48024 | AF482305 |
| *Banksia paludosa subsp. astrolux* | *Banksia paludosa* |  | JQ765219 | JQ765052 | JQ765334 |  |
| *Banksia penicillata* | *Banksia conferta* |  | AF482242 | AF482062 |  |  |
| *Banksia petiolaris* | *Banksia petiolaris* | AF482121 | AF482166 | AF482221 | AF482041 | AF482322 |
| *Banksia pilostylis* | *Banksia pilostylis* |  |  | AF482228 | AF482048 | AF482329 |
| *Banksia plagiocarpa* | *Banksia plagiocarpa* |  |  | AF482238 | AF482058 |  |
| *Banksia praemorsa* | *Banksia praemorsa* |  |  | AF482231 | AF482051 | AF482332 |
| *Banksia prionotes* | *Banksia prionotes* |  |  | AF482215 | AF482035 | AF482316 |
| *Banksia pulchella* | *Banksia pulchella* | AF482132 | AF482177 | AF482278 | AF482098 |  |
| *Banksia quercifolia* | *Banksia quercifolia* | AF482128 | AF482173 | AF482259 | AF482079 |  |
| *Banksia repens* | *Banksia repens* |  |  | AF482222 | AF482042 | AF482323 |
| *Banksia robur* | *Banksia robur* |  |  | AF482239 | AF482059 |  |
| *Banksia saxicola* | *Banksia saxicola* |  |  | AF482244 | AF482064 |  |
| *Banksia scabrella* | *Banksia scabrella* |  |  | AF482270 | AF482090 |  |
| *Banksia sceptrum* | *Banksia sceptrum* | AF482112 | AF482157 | AF482208 | AF482028 | AF482309 |
| *Banksia seminuda* | *Banksia seminuda* |  |  | AF482256 | AF482076 |  |
| *Banksia serrata* | *Banksia serrata* |  |  | AF482205 | AF482025 | AF482306 |
| *Banksia solandri* | *Banksia solandri* |  |  | AF482236 | AF482056 |  |
| *Banksia speciosa* | *Banksia speciosa* | AF482114 | AF482159 | AF482210 | AF482030 | AF482311 |
| *Banksia sphaerocarpa var. caesia* | *Banksia sphaerocarpa var. caesia* |  |  | AF482268 | AF482088 |  |
| *Banksia sphaerocarpa var. latifolia* | *Banksia sphaerocarpa var. latifolia* |  | JQ765263 | JQ765074 |  |  |
| *Banksia sphaerocarpa var. sphaerocarpa* | *Banksia sphaerocarpa var. sphaerocarpa* |  |  | AF482267 | AF482087 |  |
| *Banksia spinulosa var. collina* | *Banksia spinulosa var. collina* |  |  | AF482250 | AF482070 |  |
| *Banksia spinulosa var. cunninghamii* | *Banksia spinulosa var. cunninghamii* |  |  | AF482251 | AF482071 |  |
| *Banksia spinulosa var. neoanglica* | *Banksia spinulosa var. neoanglica* |  |  | AF482252 | AF482072 |  |
| *Banksia spinulosa var. spinulosa* | *Banksia spinulosa var. spinulosa* |  |  | AF482249 | AF482069 |  |
| *Banksia telmatiaea* | *Banksia telmatiaea* |  |  | AF482269 | AF482089 |  |
| *Banksia tricuspis* | *Banksia tricuspis* | AF482131 | AF482176 | AF482277 | AF482097 |  |
| *Banksia verticillata* | *Banksia verticillata* |  |  | AF482257 | AF482077 |  |
| *Banksia victoriae* | *Banksia victoriae* |  |  | AF482213 | AF482033 | AF482314 |
| *Banksia violacea* | *Banksia violacea* |  |  | AF482274 | AF482094 |  |
| *Dryandra anatona* | *Banksia anatona* |  |  |  | JQ765275 |  |
| *Dryandra arborea* | *Banksia arborea* |  | JQ765264 | JQ765004 | JQ765375 |  |
| *Dryandra arctotidis* | *Banksia arctotidis* |  | JQ765174 | JQ765005 |  |  |
| *Dryandra armata var. armata* | *Banksia armata* |  | JQ765175 | JQ765101 | JQ765277 | JQ765103 |
| *Dryandra armata var. ignicida* | *Banksia armata* |  | JQ765262 | JQ765095 | JQ765276 |  |
| *Dryandra baxteri* | *Banksia biterax* |  | JQ765267 | JQ765097 | JQ765281 |  |
| *Dryandra bipinnatifida subsp. bipinnatifida* | *Banksia bipinnatifida* |  | JQ765177 | JQ765008 | JQ765280 |  |
| *Dryandra bipinnatifida subsp. multifida* | *Banksia bipinnatifida* |  | JQ765176 | JQ765007 | JQ765279 | JQ765104 |
| *Dryandra blechnifolia* | *Banksia pellaeifolia* |  | JQ765220 | JQ765053 | JQ765335 |  |
| *Dryandra borealis subsp. borealis* | *Banksia borealis* |  | JQ765261 | JQ765009 | JQ765283 | JQ765106 |
| *Dryandra borealis subsp. eliator* | *Banksia borealis* |  | JQ765178 | JQ765094 | JQ765282 | JQ765105 |
| *Dryandra brownii* | *Banksia brunnea* |  | JQ765179 | JQ765010 | JQ765285 |  |
| *Dryandra calophylla* | *Banksia calophylla* | AF482135 | AF482180 | AF482281 | AF482102 | AF482338 |
| *Dryandra carlinoides* | *Banksia carlinoides* |  | JQ765180 | JQ765011 | JQ765286 | JQ765107 |
| *Dryandra catoglypta* | *Banksia catoglypta* |  | JQ765181 | JQ765012 | JQ765287 | JQ765108 |
| *Dryandra cirsioides* | *Banksia cirsoides* |  | JQ765182 | JQ765013 | JQ765288 | JQ765109 |
| *Dryandra columnaris* | *Banksia columnaris* |  |  |  | JQ765289 |  |
| *Dryandra comosa* | *Banksia comosa* |  | JQ765272 | JQ765014 | JQ765290 |  |
| *Dryandra concinna* | *Banksia concinna* |  | JQ765183 | JQ765015 | JQ765291 | JQ765110 |
| *Dryandra conferta var. conferta* | *Banksia densa* |  | JQ765190 | JQ765022 | JQ765299 | JQ765115 |
| *Dryandra conferta var. parva* | *Banksia densa* |  | JQ765189 | JQ765021 | JQ765298 | JQ765114 |
| *Dryandra corvijuga* | *Banksia corvijuga* |  |  | JQ765016 | JQ765292 |  |
| *Dryandra cuneata* | *Banksia cuneata* | AF482106 | AF482151 | AF482197 | AF482017 | AF482298 |
| *Dryandra cynaroides* | *Banksia cynaroides* |  | JQ765184 |  | JQ765293 |  |
| *Dryandra cypholoba* | *Banksia obovata* |  | JQ765215 | JQ765048 | JQ765330 | JQ765141 |
| *Dryandra drummondii subsp. drummondii* | *Banksia drummondii* |  | JQ765191 | JQ765023 | JQ765300 | JQ765116 |
| *Dryandra drummondii subsp. hiemalis* | *Banksia drummondii* |  | JQ765192 | JQ765096 | JQ765301 | JQ765117 |
| *Dryandra drummondii subsp. macrorufa* | *Banksia drummondii* |  | JQ765265 |  | JQ765302 | JQ765118 |
| *Dryandra echinata* | *Banksia echinata* |  | JQ765193 | JQ765024 | JQ765303 | JQ765119 |
| *Dryandra epimicta* | *Banksia epimicta* |  | JQ765194 | JQ765025 | JQ765304 | JQ765120 |
| *Dryandra falcata* | *Banksia falcata* |  | JQ765195 | JQ765026 | JQ765377 | JQ765121 |
| *Dryandra fasciculata* | *Banksia fasciculata* |  | JQ765196 | JQ765027 | JQ765305 | JQ765122 |
| *Dryandra ferruginea subsp. ferruginea* | *Banksia rufa* |  | JQ765234 | JQ765100 | JQ765349 | JQ765153 |
| *Dryandra ferruginea subsp. pumila* | *Banksia rufa* |  | JQ765233 | JQ765066 | JQ765348 |  |
| *Dryandra ferruginea subsp. tutanningensis* | *Banksia rufa* |  | JQ765235 | JQ765067 | JQ765350 |  |
| *Dryandra fililoba* | *Banksia fililoba* |  | JQ765197 | JQ765028 | JQ765306 | JQ765123 |
| *Dryandra foliosissima* | *Banksia foliosissima* | AF482133 | AF482178 | AF482279 | AF482100 | AF482335 |
| *Dryandra formosa* | *Banksia formosa* |  | JQ765268 | JQ765029 | JQ765307 | JQ765124 |
| *Dryandra fraseri var. ashbyi* | *Banksia fraseri* |  | JQ765273 | JQ765030 | JQ765308 | JQ765125 |
| *Dryandra fraseri var. effusa* | *Banksia fraseri* |  | JQ765198 | JQ765099 | JQ765309 | JQ765126 |
| *Dryandra fraseri var. fraseri* | *Banksia fraseri* |  | JQ765199 |  | JQ765310 | JQ765127 |
| *Dryandra fuscobractea* | *Banksia fuscobractea* |  | JQ765200 |  | JQ765311 |  |
| *Dryandra glauca* | *Banksia glaucifolia* |  | JQ765203 | JQ765034 | JQ765376 |  |
| *Dryandra hewardiana* | *Banksia hewardiana* |  | JQ765269 | JQ765098 | JQ765315 | JQ765129 |
| *Dryandra idiogenes* | *Banksia idiogenes* |  | JQ765205 | JQ765036 | JQ765316 | JQ765131 |
| *Dryandra insulanemorecincta* | *Banksia insulanemorecincta* |  | JQ765206 | JQ765037 | JQ765317 | JQ765132 |
| *Dryandra ionthocarpa* | *Banksia ionthocarpa* |  | JQ765207 | JQ765038 | JQ765318 | JQ765133 |
| *Dryandra kippistiana* | *Banksia kippistiana* |  | JQ765259 | JQ765039 | JQ765319 |  |
| *Dryandra lepidorhiza* | *Banksia lepidorhiza* |  | JQ765209 |  | JQ765321 |  |
| *Dryandra lindleyana subsp. lindleyana* | *Banksia dallaneyi* |  | JQ765185 | JQ765017 | JQ765294 | JQ765111 |
| *Dryandra lindleyana subsp. lindleyana var. mellicula* | *Banksia dallaneyi* |  | JQ765187 | JQ765019 | JQ765296 | JQ765113 |
| *Dryandra lindleyana subsp. pollosa* | *Banksia dallaneyi* |  | JQ765188 | JQ765020 | JQ765297 |  |
| *Dryandra lindleyana subsp. silvestris* | *Banksia dallaneyi* |  | JQ765186 | JQ765018 | JQ765295 | JQ765112 |
| *Dryandra longifolia subsp. archeos* | *Banksia prolata* |  | JQ765226 | JQ765059 | JQ765341 | JQ765149 |
| *Dryandra longifolia subsp. calcicola* | *Banksia prolata* |  | JQ765227 | JQ765060 | JQ765342 | JQ765150 |
| *Dryandra longifolia subsp. longifolia* | *Banksia prolata* |  | JQ765228 | JQ765061 | JQ765343 | JQ765151 |
| *Dryandra meganotia* | *Banksia meganoita* |  |  | JQ765041 | JQ765322 |  |
| *Dryandra mimica* | *Banksia mimica* |  | JQ765210 | JQ765042 | JQ765323 | JQ765135 |
| *Dryandra mucronulata subsp. retrorsa* | *Banksia mucronulata* |  | JQ765266 | JQ765043 | JQ765324 | JQ765136 |
| *Dryandra nana* | *Banksisa nana* |  |  |  | JQ765325 |  |
| *Dryandra nervosa* | *Banksia alliacea* |  | JQ765173 | JQ765003 | JQ765274 | JQ765102 |
| *Dryandra nivea subsp. fuliginosa* | *Banksia nivea subsp. fuliginosa* |  | JQ765211 | JQ765044 | JQ765326 | JQ765137 |
| *Dryandra nivea subsp. nivea* | *Banksia nivea subsp. nivea* |  | JQ765212 | JQ765045 | JQ765327 | JQ765138 |
| *Dryandra nobilis subsp. fragrans* | *Banksia nobilis subsp. fragrans* |  | JQ765213 | JQ765046 | JQ765328 | JQ765139 |
| *Dryandra nobilis subsp. nobilis* | *Banksia nobilis subsp. nobilis* |  | JQ765214 | JQ765047 | JQ765329 | JQ765140 |
| *Dryandra obtusa* | *Banksia obtusa* |  | JQ765216 | JQ765049 | JQ765331 | JQ765142 |
| *Dryandra octotriginta* | *Banksia octotriginta* |  | JQ765217 | JQ765050 | JQ765332 | JQ765143 |
| *Dryandra pallida* | *Banksia pallida* |  | JQ765218 | JQ765051 | JQ765333 | JQ765144 |
| *Dryandra platycarpa* | *Banksia platycarpa* |  | JQ765221 | JQ765054 | JQ765336 | JQ765145 |
| *Dryandra plumosa subsp. denticulata* | *Banksia plumosa* |  | JQ765222 | JQ765055 | JQ765337 | JQ765146 |
| *Dryandra polycephala* | *Banksia polycephala* |  | JQ765223 | JQ765056 | JQ765338 | JQ765147 |
| *Dryandra porrecta* | *Banksia porrecta* |  | JQ765224 | JQ765057 |  | JQ765148 |
| *Dryandra praemorsa var. praemorsa* | *Banksia undata* |  | JQ765255 | JQ765090 | JQ765370 | JQ765168 |
| *Dryandra praemorsa var. splendens* | *Banksia undata* |  | JQ765254 | JQ765089 | JQ765369 | JQ765167 |
| *Dryandra prionotes* | *Banksia prionophylla* |  | JQ765225 | JQ765058 | JQ765339 |  |
| *Dryandra proteoides* | *Banksia proteoides* |  |  |  | JQ765340 |  |
| *Dryandra pseudoplumosa* | *Banksia pseudoplumosa* |  | JQ765229 | JQ765062 | JQ765344 |  |
| *Dryandra pteridifolia subsp. incretita* | *Banksia pteridifolia* |  | JQ765231 | JQ765064 | JQ765346 | JQ765152 |
| *Dryandra pteridifolia subsp. vernalis* | *Banksia pteridifolia* |  | JQ765230 | JQ765063 | JQ765345 |  |
| *Dryandra pulchella* | *Banksia bella* |  |  | JQ765006 | JQ765278 |  |
| *Dryandra purdieana* | *Banksia purdieana* |  | JQ765232 | JQ765065 | JQ765347 |  |
| *Dryandra quercifolia* | *Banksia heliantha* |  | JQ765204 | JQ765035 | JQ765314 |  |
| *Dryandra rufistylis* | *Banksia rufistylis* |  | JQ765236 | JQ765068 | JQ765351 |  |
|  | *Banksia rosserae* |  | JQ765271 |  |  |  |
| *Dryandra serra* | *Banksia serra* |  | JQ765237 | JQ765069 | JQ765352 |  |
| *Dryandra serratuloides subsp. perissa* | *Banksia serratuloides subsp. perissa* | AF482134 | AF482179 | AF482280 | AF482101 | AF482337 |
| *Dryandra sessilis var. cordata* | *Banksia sessilis var. cordata* |  | JQ765238 | JQ765070 | JQ765353 |  |
| *Dryandra sessilis var. cygnorum* | *Banksia sessilis var. cygnorum* | AF482136 | AF482181 | AF48282 | AF482103 | AF482339 |
| *Dryandra sessilis var. sessilis* | *Banksia sessilis var. sessilis* |  | JQ765239 | JQ765071 | JQ765354 |  |
| *Dryandra shanklandiorum* | *Banksia shanklandiorum* |  | JQ765240 | JQ765072 | JQ765355 | JQ765154 |
| *Dryandra shuttleworthia* | *Banksia shuttleworthia* |  | JQ765241 | JQ765073 | JQ765356 | JQ765155 |
| *Dryandra speciosa* | *Banksia splendida* | AF482137 | AF482182 | AF482283 | AF482104 | AF482340 |
| *Dryandra speciosa subsp. macrocarpa* | *Banksia splendida subsp. macrocarpa* |  | JQ765242 | JQ765075 | JQ765357 | JQ765156 |
| *Dryandra speciosa subsp. speciosa* | *Banksia splendida subsp. splendida* |  | JQ765243 | JQ765076 |  | JQ765157 |
| *Dryandra squarrosa subsp. argillacea* | *Banksia squarrosa subsp. argillacea* |  | JQ765244 | JQ765077 | JQ765358 | JQ765158 |
| *Dryandra squarrosa subsp. squarrosa* | *Banksia squarrosa subsp. squarrosa* |  | JQ765270 | JQ765078 | JQ765359 | JQ765159 |
| *Dryandra stenoprion* | *Banksia stenoprion* |  | JQ765245 | JQ765079 |  | JQ765160 |
| *Dryandra stricta* | *Banksia strictifolia* |  | JQ765246 | JQ765080 | JQ765360 |  |
| *Dryandra stuposa* | *Banksia stuposa* |  | JQ765247 | JQ765081 | JQ765361 | JQ765161 |
| *Dryandra subpinnatifida var. imberbis* | *Banksia subpinnatifida var. imberbis* |  | JQ765248 | JQ765082 | JQ765362 | JQ765162 |
| *Dryandra subpinnatifida var. subpinnatifida* | *Banksia subpinnatifida var. subpinnatifida* |  | JQ765249 | JQ765083 | JQ765363 | JQ765163 |
| *Dryandra subulata* | *Banksia subulata* |  |  | JQ765084 | JQ765364 |  |
| *Dryandra tenuifolia var. reptans* | *Banksia tenuis var. reptans* |  | JQ765250 | JQ765085 | JQ765365 | JQ765164 |
| *Dryandra tenuifolia var. tenuifolia* | *Banksia tenuis var. tenuifolia* |  | JQ765251 | JQ765086 | JQ765366 | JQ765165 |
| *Dryandra tortifolia* | *Banksia tortifolia* |  | JQ765252 | JQ765087 | JQ765367 | JQ765166 |
| *Dryandra tridentata* | *Banksia tridentata* |  | JQ765253 | JQ765088 | JQ765368 |  |
| *Dryandra vestita* | *Banksia vestita* |  | JQ765256 | JQ765091 | JQ765371 | JQ765169 |
| *Dryandra viscida* | *Banksia viscida* |  | JQ765257 | JQ765092 | JQ765372 | JQ765170 |
| *Dryandra wonganensis* | *Banksia wonganensis* |  | JQ765258 | JQ765093 | JQ765373 | JQ765171 |
