# Supplementary material for: Evolution of a hotspot genus: geographic variation in speciation and extinction rates in Banksia (Proteaceae)
Source: BMC Evol Biol. 2013 Aug 19;13:155. doi: 10.1186/1471-2148-13-155 (PMC3751403; doi:10.1186/1471-2148-13-155)
Supplement: Additional file 2 — Chloroplast regions amplified and primer sequences. [file 1471-2148-13-155-S2.docx]

**Additional File 2. Chloroplast regions amplified and primer sequences.**

Cardillo, M. & Pratt, R. Evolution of a hotspot genus: geographic variation in speciation and extinction rates in *Banksia* (Proteaceae)

| Region | Primer | Sequence 5'-3' | Reference |
| --- | --- | --- | --- |
| *rpl16* intron | F71 | GCTATGCTTAGTGTGTGACTCGTTG | Jordan, Courtney & Neigel 1996 |
|  | R1516 | CCCTTCATTCTTCCTCTATGTTG | Baum, Small & Wendel 1998 |
|  |  |  |  |
| *psbA/trnH* spacer | psbAF | GTTATGCATGAACGTAATGCTC | Sang, Crawford & Stuessy 1997 |
|  | trnHR | CGCGCATGGTGGATTCACAAATC | Sang, Crawford & Stuessy 1997 |
|  |  |  |  |
| *trnT-trnL* spacer | a | CATTACAAATGCGATGCTCT | Taberlet, Gielly, Pautou & Bouvet 1991 |
|  | b | TCTACCGATTTCGCCATATC | Taberlet, Gielly, Pautou & Bouvet 1991 |
|  |  |  |  |
| *trnL-trnF* intergenic spacer | c | CGAAATCGGTAGACGCTACG | Taberlet, Gielly, Pautou & Bouvet 1991 |
|  | d | GGGGATAGAGGGACTTGAAC | Taberlet, Gielly, Pautou & Bouvet 1991 |
|  | e | GGTTCAAGTCCCTCTATCCC | Taberlet, Gielly, Pautou & Bouvet 1991 |
|  | f | ATTTGAACTGGTGACACGAG | Taberlet, Gielly, Pautou & Bouvet 1991 |

**Literature Cited**

Taberlet, P., L. Gielly, G. Pautou, and J. Bouvet. 1991. Universal primers for amplification of three non-coding regions of chloroplast DNA. Plant Mol. Biol. 17:1105–1109.

Jordan, W. C., Courtney, M. W. & Neigel, J. E. 1996. Low levels of intraspecific genetic variation at a rapidly evolving chloroplast DNA locus in North American Duckweeds (Lemnaceae), American Journal of Botany, 83(4): 430-439

Baum, D. A., Small, R. L. & Wendel, J. F. 1998. Biogeoraphy and floral evolution of Boababs (Adansonia, Bombacaceae) as inferred from multiple data sets. Syst. Biol. 47(2): 181-207

Sang, T., Crawford, D. J., & Stuessy, T. F. 1997. Chloroplast DNA phylogeny, reticulate evolution, and biogeography of Paeonia (Paeoniaceae), American Journal of Botany, 84 (9): 1120-1136
